# Genetic Polymorphisms in Host Innate Immune Sensor Genes and the Risk of Nasopharyngeal Carcinoma in North Africa

**DOI:** 10.1534/g3.112.005371

**Published:** 2013-06-01

**Authors:** Khalid Moumad, Jesus Lascorz, Melanie Bevier, Meriem Khyatti, Moulay Mustapha Ennaji, Abdellatif Benider, Stefanie Huhn, Shun Lu, Lotfi Chouchane, Marilys Corbex, Kari Hemminki, Asta Försti

**Affiliations:** * Department of Molecular Genetic Epidemiology, German Cancer Research Center (DKFZ), 69120 Heidelberg, Germany; † Oncovirology Laboratory, Institut Pasteur du Maroc, 20360 Casablanca, Morocco; ‡ Laboratory of Virology, Hygiene & Microbiology, Faculty of Sciences & Technics, University Hassan II Mohammedia-Casablanca, 20650 Mohammedia, Morocco; § Service de Radiothérapie, Centre d’oncologie Ibn Rochd, 9155 Casablanca, Morocco; ** Genetic Medicine and Immunology Laboratory, Weill Cornell Medical College in Qatar, Qatar Foundation, Education City, 24144 Doha, Qatar; †† Department of Public Health, Institute of Tropical Medicine, 2000 Antwerp, Belgium; ‡‡ Center of Primary Health Care Research, Clinical Research Center, Lund University, SE-20502 Malmö, Sweden

**Keywords:** nasopharyngeal carcinoma, North Africa, host innate immune sensors, SNPs, Epstein-Barr virus

## Abstract

Nasopharyngeal carcinoma (NPC) is a rare malignancy in most parts of the world. It is an Epstein-Barr virus−associated malignancy with an unusual racial and geographical distribution. The host innate immune sensor genes play an important role in infection recognition and immune response against viruses. Therefore, we examined the association between polymorphisms in genes within a group of pattern recognition receptors (including families of Toll-like receptors, C-type lectin receptors, and retinoic acid−inducible gene I−like receptors) and NPC susceptibility. Twenty-six single-nucleotide polymorphisms (SNPs) in five pattern-recognition genes were genotyped in 492 North African NPC cases and 373 frequency-matched controls. TLR3_rs3775291 was the most significantly associated SNP (odds ratio [OR] 1.49; 95% confidence interval [95% CI] 1.11−2.00; *P* = 0.008; dominant model). The analysis showed also that CD209_rs7248637 (OR 0.69; 95% CI 0.52−0.93; *P* = 0.02; dominant model) and DDX58_rs56309110 (OR 0.70; 95% CI 0.51−0.98; *P* = 0.04) were associated with the risk of NPC. An 18% increased risk per allele was observed for the five most significantly associated SNPs, TLR3_rs3775291, CD209_rs7248637, DDX58_rs56309110, CD209_rs4804800, and MBL2_rs10824792, (p_trend_ = 8.2 × 10^−4^). Our results suggest that genetic variation in pattern-recognition genes is associated with the risk of NPC. These preliminary findings require replication in larger studies.

Nasopharyngeal carcinoma (NPC) is a highly invasive and metastatic malignant tumor that occurs in the epithelial cells lining the nasopharynx and shows a distinct geographical distribution. It is uncommon among white residents in Western Europe and North America, with an age-adjusted incidence for both sexes less than 1/100,000, whereas the greatest rates are reported among Cantonese in Southern China and intermediate rates in other regions, such as North Africa. The age-adjusted incidence for both sexes reach 25/100,000 in South East Asia and 5/100,000 in North Africa (Busson *et al.* 2004). Epstein-Barr virus (EBV), a gammaherpesvirus, is consistently associated with the World Health Organization type II and III NPC, irrespective of ethnic origin or geographical distribution. Despite the fact that EBV infection is ubiquitous worldwide, the development of NPC remains confined in a subset of infected population, suggesting that there are other factors contributing to the development of NPC (Busson *et al.* 2004). Indeed, studies on EBV-associated tumors have suggested specific interactions between environmental, genetic, and viral factors (Feng *et al.* 2009; Jia and Qin 2012). The observation that Chinese emigrants from endemic areas continue to have a high incidence of NPC, regardless of their country of immigration (Chang and Adami 2006), also suggests that genetic factors, such as single-nucleotide polymorphisms (SNPs), may play a role in the susceptibility of this disease.

During viral infection, innate immunity is the first line of defense. It orchestrates host responses to prevent or reduce viral replication and spread until the adaptive immune system is operational and able to eliminate the specific invading pathogen and to generate immunological memory. Cellular viral sensors have long been recognized as crucial mediators of innate antiviral defense with important effects on the magnitude and quality of both innate and adaptive immune responses. Important antiviral factors and pathways, such as the retinoic acid-inducible gene I protein/DEAD (Asp-Glu-Ala-Asp) box polypeptide 58 (RIG-I/DDX58), Toll-like receptors (TLRs), mannose-binding lectin (encoded by *MBL2*), and dendritic cell−specific intercellular adhesion molecule-3−grabbing non-integrin (DC-SIGN, also known as CD209) play a role in viral sensing, control, pathogenesis, and outcome of viral infections (Thompson and Iwasaki 2008; Nakhaei *et al.* 2009; Faure and Rabourdin-Combe 2011; Frakking *et al.* 2011; Clingan *et al.* 2012).

Inherited polymorphisms in cellular viral sensor genes are potential determinants of immune response heterogeneity that may influence the immune responses by altering the functionality and antiviral effects of the corresponding proteins. Genetic variants in these genes have been implicated as important regulators of immunity and host response to infection and to malignancies (El-Omar *et al.* 2008; Haralambieva *et al.* 2011).

Bearing in mind the multifaceted interactions between viruses and factors of the innate immune system, we sought to investigate the role of cellular antiviral sensors as plausible contributors to immune response heterogeneity in the development of NPC. For this reason, we performed a comprehensive candidate gene association study to investigate the role of potentially functional SNPs located within the *CD209*, *DDX58*, *MBL2*, *TLR2*, *TLR3*, and *TLR9* genes on the risk of NPC.

## Materials and Methods

### Study population

Details of the studied populations are described elsewhere (Feng *et al.* 2007, 2009). In brief, 333 NPC cases and 373 controls were recruited between the years 2001 and 2004 from four centers located in two North African countries with a high incidence of NPC: Morocco (Casablanca and Rabat) and Tunisia (Tunis and Sousse). An additional 159 NPC cases from Casablanca, Morocco, recruited between the years 2006 and 2009 were added in the current study. Inclusion criteria stipulated that all four grandparents of each subject were of Moroccan or Tunisian origin. The hospital-based controls were cancer-free individuals and unrelated to the patients. They were matched to the NPC cases by sex, age, and childhood household type (rural or urban). At recruitment, informed consent was obtained from each subject, who was then interviewed to collect detailed information on demographic characteristics. The baseline characteristics of the population sample analyzed in our study are shown in Table 1. The study was approved by the International Agency for Research on Cancer ethical committee.

**Table 1:** Basic characteristics of the study population

	Cases, n (%)	Controls, n (%)	*P* Value*^a^*
Whole population	492	373	
Gender			
Male	357 (72.56)	246 (65.95)	0.04
Female	135 (27.44)	127 (34.05)	
Age, median (range)	43 (10-89)	42 (14-85)	0.10
Moroccan population	309	210	
Gender			
Male	224 (72.49)	143 (68.10)	0.28
Female	85 (27.51)	67 (31.90)	
Age, median (range)	43 (12-89)	41.5 (14-85)	0.34
Tunisian population	183	163	
Gender			
Male	133 (72.68)	103 (63.19)	0.06
Female	50 (27.32)	60 (36.81)	
Age, median (range)	42 (10-76)	44 (14-75)	0.16

a Difference tested with Wilcoxon rank sum test.

### SNP selection

A total of 26 SNPs across six innate immune genes (*CD209*, *DDX58*, *MBL2*, *TLR2*, *TLR3*, and *TLR9*) were selected to the study based on data obtained from the International HapMap Project (http://hapmap.ncbi.nlm.nih.gov) and the NCBI database (http://www.ncbi.nlm.nih.gov) for the CEU (Utah residents with Northern and Western European ancestry from the CEPH collection) and the YRI (Yoruba in Ibadan, Nigeria) populations, as no information was available for any Northern African population (Bosch *et al.* 2001; Hajjej *et al.* 2006). The selection criteria were as follows: (1) minor allele frequency ≥10%; (2) location within the coding region (nonsynonymous SNPs), the 3′ and 5′ untranslated regions (UTRs), and the promoter (up to approximately 1 kb from the transcription start site); and (3) linkage disequilibrium (LD; r^2^ < 0.80) between the SNPs. We explored the potential function of the associated SNPs as well as other potential causal variants in LD (r^2^ ≥ 0.80) with these SNPs using FuncPred (http://snpinfo.niehs.nih.gov/index.html). The SNPs selected to the study are shown in Table 2.

**Table 2:** Selected SNPs

Gene	SNP	Chr.	Position	Allele	Location	TFBS*^a^*	miRNA*^a^*	nsSNP	Aa Change	Polyphen*^a^*
CD209	rs2287886	19	7718536	A/G	Promoter	+	–	–	–	–
CD209	rs4804803	19	7718733	A/G	Promoter	+	–	–	–	–
CD209	rs735240	19	7719336	A/G	Promoter	+	–	–	–	–
CD209	rs4804800	19	7711128	A/G	3′-UTR	+	+	–	–	–
CD209	rs11465421	19	7711296	T/G	3′-UTR	+	–	–	–	–
CD209	rs7248637	19	7713027	A/G	3′-UTR	–	+	–	–	–
DDX58	rs56309110	9	32516754	G/T	Promoter	+	–	–	–	–
DDX58	rs1133071	9	32445674	G/A	3′-UTR	–	+	–	–	–
DDX58	rs12006123	9	32446017	A/G	3′-UTR	–	+	–	–	–
DDX58	rs7029002*^b^*	9	32445320	C/T	3′-UTR	–	+	–	–	–
DDX59	rs10813831	9	32516146	A/G	Exon	–	–	+	R7C	Probably damaging
DDX58	rs17217280	9	32470251	A/T	Exon	–	–	+	D580E	Benign
DDX58	rs3739674	9	32516233	G/C	5′-UTR	+	–	–	–	–
MBL2	rs11003125	10	54202020	C/G	Promoter	+	–	–	–	–
MBL2	rs7096206	10	54201691	C/G	Promoter	+	–	–	–	–
MBL2	rs920724	10	54202803	A/G	Promoter	+	–	–	–	–
MBL2	rs10824792	10	54196212	C/T	3′-UTR	–	+	–	–	–
MBL2	rs2083771	10	54195684	G/T	3′-UTR	–	+	–	–	–
MBL2	rs1800450*^c^*	10	54201241	T/C	Exon	–	–	+	G54N	Probably damaging
TLR2	rs5743704*^d^*	4	154845401	A/C	Exon	–	–	+	P631H	Probably damaging
TLR2	rs5743708*^d^*	4	154845767	A/G	Exon	–	–	+	R753Q	Possibly damaging
TLR3	rs3775291	4	187241068	T/C	Exon	–	–	+	L412F	Possibly damaging
TLR9	rs187084	3	52236071	G/A	Promoter	+	–	–	–	–
TLR9	rs352139	3	52233412	T/C	Promoter	–	–	–	–	–
TLR9	rs5743836	3	52235822	G/A	Promoter	+	–	–	–	–
TLR9	rs5743840*^d^*	3	52235252	T/A	Promoter	+	–	–	–	–

SNP, single-nucleotide polymorphism; Chr., chromosome; TFBS, transcription factor-binding site; nsSNP, non-synonymous coding SNP; Aa, amino acid; UTR, untranslated region.

a FuncPred tool was used to predict the functional consequences of the SNPs: +, positive prediction; –, no prediction.

b Assay failed.

c Genotype frequencies in controls were not in HWE and the SNP was excluded from the analyses.

d Monomorphic SNP.

### Genotyping

High-quality genomic DNA was available for 492 NPC cases and 373 controls from Morocco and Tunisia. Genotyping was performed using KASPar SNP Genotyping system (KBioscience, Hoddesdon, UK) in a 384-well plate format. Polymerase chain reaction products were analyzed with the ABI Prism 7900HT detection system using the SDS 2.4 software (Applied Biosystems, Foster City, CA). Internal quality controls (approximately 10% of samples randomly selected and included as duplicate) showed >99% concordance for each assay. The mean call rate was 97%.

### Statistical analysis

The observed genotype frequencies in controls were tested for Hardy-Weinberg equilibrium using a Pearson goodness-of-fit test (http://ihg2.helmholtz-muenchen.de/cgi-bin/hw/hwa1.pl). The most common genotype in the control group was assigned as the reference category and odds ratios (ORs) and their corresponding 95% confidence intervals (CIs) were estimated using multiple logistic regressions after inclusion of matching variables (center, age, and sex). All tests were considered to be statistically significant with a *P* < 0.05. Estimates of pair-wise LD based on the r-squared statistic were obtained using Haploview software, version 4.2. Haplotype block structure was determined using the method of Gabriel *et al.* (2002) with the HaploView software and the SNPtool (http://www.dkfz.de/de/molgen_epidemiology/tools/SNPtool.html).

Cumulative impact of the alleles that were nominally associated with the risk of NPC (*P* < 0.10) in the present study was evaluated by counting one for a heterozygous genotype and two for a homozygous genotype. Epistasis between all studied SNPs was tested using multifactor dimensionality reduction (MDR) method for interaction (Ritchie *et al.* 2001). This model-free, nonparametric data reduction method classifies multilocus genotypes into high-risk and low-risk groups. The MDR version 2.0 beta 5 with the MDRpt version 0.4.9 alpha module for permutation testing is an open-source and freely available software (http://www.epistasis.org/). The software estimates the importance of the signals by using both cross-validation and permutation testing, which generates an empirical p-value for the result. A *P* < 0.05 was considered statistically significant.

## Results

From the 26 originally selected SNPs, three (TLR2_rs5743704, TLR2_rs5743708, and TLR9_rs5743840) turned out to be monomorphic in our North African study population, and one failed genotyping (DDX58_rs7029002). Genotype frequencies and LD patterns did not differ significantly between the two countries (Table 3 and Supporting Information, Figure S1) (Hajjej *et al.* 2006). The genotype frequencies in controls were in Hardy-Weinberg equilibrium with the exception of MBL2_rs1800450 (*P* = 0.001). The SNP was excluded from further analyses.

**Table 3:** Single-locus association analyses

Gene name_rs	M/m	Call Rate	Population	Genotypes (MM-Mm-mm)		Dominant Model (Without Covariates)		Dominant Model (With Covariates*^a^*)		P_HWE_*^b^*
				Controls	Cases	OR (95% CI)	*P* Value	OR (95% CI)	*P* Value	
CD209_rs2287886	G/A	0.98	All	194–134–39	237–197–46	1.15 (0.88–1.51)	0.31	1.12 (0.85–1.47)	0.42	0.03
			Morocco	107–76–24	153–128–19	1.03 (0.72–1.47)	0.88	0.99 (0.69–1.42)	0.97	
			Tunisia	87–58–15	84–69–27	1.36 (0.89–2.09)	0.16	1.35 (0.88–2.08)	0.17	
CD209_rs4804800	A/G	0.98	All	237–115–13	344–127–14	0.76 (0.57–1.02)	0.06	0.75 (0.56–1.01)	0.05	0.84
			Morocco	132–67–8	219–75–10	0.68 (0.47–1.00)*^C^*	0.048*^C^*	0.69 (0.47–1.00)	0.05	
			Tunisia	105–48–5	125–52–4	0.89 (0.56–1.40)	0.61	0.85 (0.53–1.35)	0.48	
CD209_rs4804803	A/G	0.90	All	185–125–29	245–162–33	0.96 (0.72–1.27)	0.76	0.93 (0.70–1.24)	0.64	0.26
			Morocco	102–70–17	147–113–17	1.04 (0.72–1.50)	0.85	1.06 (0.73–1.53)	0.77	
			Tunisia	83–55–12	98–49–16	0.82 (0.52–1.29)	0.39	0.74 (0.47–1.18)	0.21	
CD209_rs735240	C/T	0.98	All	122–173–72	145–244–91	1.15 (0.86–1.54)	0.35	1.16 (0.86–1.56)	0.32	0.45
			Morocco	70–98–39	80–165–55	1.41 (0.96–2.07)	0.08	1.43 (0.97–2.11)	0.07	
			Tunisia	52–75–33	65–79–36	0.85 (0.54–1.34)	0.48	0.88 (0.56–1.38)	0.56	
CD209_rs11465421	A/C	0.99	All	119–183–70	158–242–86	0.98 (0.73–1.30)	0.87	0.95 (0.71–1.27)	0.73	0.94
			Morocco	59–111–39	105–152–49	0.75 (0.51–1.10)	0.15	0.74 (0.50–1.08)	0.12	
			Tunisia	60–72–31	53–90–37	1.40 (0.89–2.19)	0.15	1.38 (0.87–2.17)	0.18	
CD209_rs7248637	G/A	0.93	All	215–118–14	320–127–13	0.71 (0.53–0.96)*^C^*	0.02*^C^*	0.69 (0.52–0.93)*^C^*	0.02*^C^*	0.67
			Morocco	117–70–9	209–75–6	0.57 (0.39–0.84)*^C^*	0.005*^C^*	0.57 (0.39–0.84)*^C^*	0.004*^C^*	
			Tunisia	98–48–5	111–52–7	0.98 (0.62–1.56)	0.94	0.90 (0.56–1.44)	0.66	
DDX58_rs56309110	G/T	0.97	All	272–91–2	386–82–8	0.69 (0.50–0.96)*^C^*	0.03*^C^*	0.70 (0.51–0.98)*^C^*	0.04*^C^*	0.05
			Morocco	158–45–1	248–47–5	0.72 (0.46–1.12)	0.15	0.71 (0.45–1.11)	0.13	
			Tunisia	114–46–1	138–35–3	0.69 (0.42–1.12)	0.13	0.70 (0.43–1.15)	0.16	
DDX58_rs1133071	T/C	0.98	All	177–156–35	258–187–36	0.80 (0.61–1.05)	0.11	0.81 (0.61–1.06)	0.12	0.92
			Morocco	104–90–15	166–111–23	0.80 (0.56–1.14)	0.22	0.80 (0.56–1.14)	0.22	
			Tunisia	73–66–20	92–76–13	0.82 (0.54–1.26)	0.37	0.81 (0.53–1.25)	0.34	
DDX58_rs12006123	G/A	0.95	All	261–88–7	357–105–8	0.87 (0.63–1.19)	0.39	0.90 (0.65–1.24)	0.51	0.90
			Morocco	155–45–1	229–64–4	1.00 (0.65–1.53)	1.00	1.02 (0.66–1.56)	0.95	
			Tunisia	105–43–6	128–41–4	0.76 (0.47–1.23)	0.26	0.76 (0.47–1.24)	0.27	
DDX58_rs10813831	G/A	0.99	All	242–107–21	298–168–20	1.19 (0.90–1.58)	0.22	1.21 (0.91–1.60)	0.20	0.05
			Morocco	138–60–12	192–98–15	1.13 (0.78–1.63)	0.52	1.14 (0.79–1.65)	0.49	
			Tunisia	104–47–9	70–106–5	1.31 (0.85–2.04)	0.22	1.30 (0.83–2.03)	0.25	
DDX58_rs17217280	A/T	0.97	All	247–110–10	315–153–10	1.07 (0.80–1.42)	0.67	1.06 (0.79–1.41)	0.72	0.59
			Morocco	140–63–4	196–96–8	1.11 (0.76–1.62)	0.59	1.12 (0.77–1.64)	0.54	
			Tunisia	107–47–6	119–57–2	1.00 (0.64–1.58)	1.00	0.95 (0.60–1.50)	0.82	
DDX58_rs3739674	G/C	0.96	All	143–165–55	174–218–73	1.09 (0.82–1.44)	0.56	1.10 (0.83–1.47)	0.50	0.52
			Morocco	77–96–32	113–132–50	0.97 (0.67–1.40)	0.87	0.98 (0.68–1.42)	0.91	
			Tunisia	66–69–23	61–86–23	1.28 (0.82–2.00)	0.27	1.33 (0.85–2.08)	0.22	
MBL2_rs11003125	G/C	0.97	All	220–130–18	288–160–28	0.97 (0.74–1.28)	0.83	0.99 (0.75–1.31)	0.92	0.84
			Morocco	130–68–10	182–91–23	1.04 (0.72–1.51)	0.82	1.04 (0.72–1.51)	0.82	
			Tunisia	90–62–8	106–69–5	0.90 (0.58–1.38)	0.62	0.92 (0.60–1.42)	0.71	
MBL2_rs7096206	C/G	0.97	All	261–91–10	354–108–11	0.87 (0.64–1.18)	0.37	0.89 (0.65–1.22)	0.47	0.58
			Morocco	155–42–7	218–69–5	1.07 (0.71–1.63)	0.74	1.10 (0.73–1.68)	0.65	
			Tunisia	106–49–3	136–39–6	0.67 (0.42–1.08)	0.10	0.67 (0.42–1.08)	0.10	
MBL2_rs920724	A/G	0.98	All	146–175–48	183–215–81	1.06 (0.80–1.40)	0.69	1.04 (0.78–1.38)	0.80	0.69
			Morocco	74–101–32	124–127–51	0.80 (0.55–1.15)	0.23	0.78 (0.54–1.12)	0.18	
			Tunisia	72–74–16	59–88–30	1.60 (1.03–2.49)*^C^*	0.04*^C^*	1.59 (1.02–2.48)*^C^*	0.04*^C^*	
MBL2_rs10824792	C/T	0.98	All	125–175–68	135–232–112	1.31 (0.98–1.76)	0.07	1.33 (0.99–1.78)	0.06	0.62
			Morocco	72–94–42	91–144–66	1.22 (0.84–1.78)	0.30	1.24 (0.85–1.81)	0.27	
			Tunisia	53–81–26	44–88–46	1.51 (0.94–2.42)	0.09	1.49 (0.92–2.40)	0.10	
MBL2_rs2083771	T/G	0.97	All	149–172–45	211–220–42	0.85 (0.64–1.12)	0.26	0.82 (0.62–1.09)	0.17	0.69
			Morocco	76–102–29	119–144–31	0.85 (0.59–1.23)	0.40	0.84 (0.58–1.22)	0.37	
			Tunisia	73–70–16	92–76–11	0.80 (0.52–1.23)	0.31	0.79 (0.51–1.22)	0.29	
TLR3rs3775291	G/A	0.96	All	252–96–14	289–170–13	1.45 (1.09–1.94)*^C^*	0.01*^C^*	1.49 (1.11–2.00)*^C^*	0.008*^C^*	0.21
			Morocco	140–56–8	177–107–8	1.42 (0.97–2.07)	0.07	1.46 (1.00–2.14)*^C^*	0.05*^C^*	
			Tunisia	112–40–6	112–63–5	1.48 (0.94–2.33)	0.09	1.53 (0.96–2.43)	0.07	
TLR9_rs187084	T/C	0.97	All	149–177–36	212–193–69	0.87 (0.66–1.14)	0.30	0.85 (0.64–1.13)	0.26	0.12
			Morocco	85–98–21	143–111–41	0.76 (0.53–1.09)	0.13	0.75 (0.52–1.07)	0.11	
			Tunisia	64–79–15	69–82–28	1.09 (0.70–1.68)	0.71	1.03 (0.66–1.61)	0.89	
TLR9_rs352139	A/G	0.94	All	86–186–77	139–212–114	0.77 (0.56–1.05)	0.10	0.79 (0.58–1.09)	0.15	0.23
			Morocco	54–98–48	93–128–69	0.84 (0.56–1.26)	0.40	0.85 (0.57–1.26)	0.41	
			Tunisia	32–88–39	46–84–45	0.71 (0.42–1.18)	0.18	0.72 (0.43–1.20)	0.21	
TLR9_rs5743836	T/C	0.98	All	251–104–11	354–113–13	0.78 (0.58–1.05)	0.10	0.81 (0.60–1.10)	0.18	0.95
			Morocco	150–51–5	219–74–8	1.00 (0.67–1.49)	0.99	1.01 (0.68–1.51)	0.95	
			Tunisia	101–53–6	135–39–5	0.56 (0.35–0.89)*^C^*	0.01*^C^*	0.61 (0.38–0.97)*^C^*	0.04*^C^*	

M/m, Major/minor alleles. OR, odds ratio; CI, confidence interval.

a Adjusted for age, gender, and center for “all” and for age and gender for the individual analyses of the Moroccan and the Tunisian population.

b Hardy-Weinberg equilibrium *P*-values for tests of deviations from Hardy-Weinberg equilibrium in the controls.

c Indicate a statistical significance at 5% level.

In the pooled population, three SNPs were significantly associated with the risk of NPC (Table 3). The strongest association was observed for TLR3_rs3775291; the A-allele carriers had an increased risk of NPC with an OR of 1.49 (95% CI 1.11−2.00, *P* = 0.008). Additionally, the minor allele carriers of the SNPs CD209_rs7248637 and DDX58_rs56309110 had a decreased risk of NPC (OR 0.69 95% CI 0.52−0.93 and OR 0.70 95% CI 0.51−0.98, respectively). Considering the number of statistical tests (21 SNPs analyzed for the dominant model), none of the associations did survive the conservative Bonferroni correction (*P* = 0.05/21 = 0.002). However, for *TLR3* and *DDX58*, the ORs for the Moroccan and Tunisian populations were almost identical, showing internal consistency in the results.

**Figure 1:**
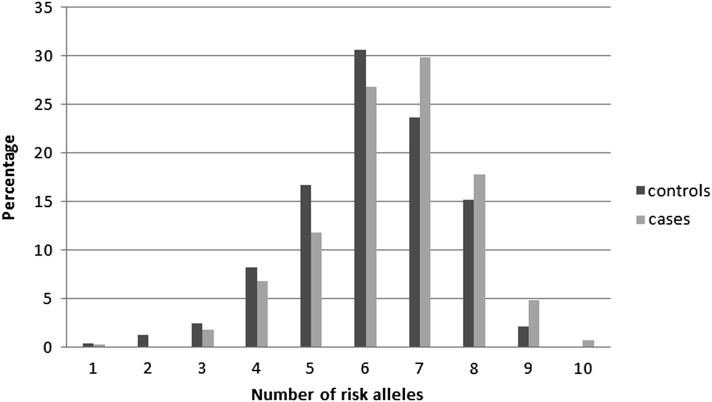
Distributions of the risk alleles by disease status (risk alleles: TLR3_rs3775291, DDX58_rs56309110, MBL2_rs10824792, CD209_rs4804800, and CD209_rs7248637).

Figure 1 shows the case and control distribution according to the cumulative number of risk alleles. Combining genotypes of the five most significantly associated SNPs (*P* < 0.10) for the 419 cases and 331 controls, we calculated ORs corresponding to an increasing number of risk alleles. The risk of NPC increased significantly, with a per-allele OR of 1.18, 95% CI 1.07–1.29 (p_trend_ = 8.2 × 10^−4^). For carriers of more than six risk alleles, the risk of disease was increased 1.64-fold (OR 1.64, 95% CI 1.22–2.19, *P* = 9.0 × 10^−4^), compared with carriers of less than or equal to six risk alleles. We also analyzed high-order interactions between SNPs using the MDR algorithm. No combination of possibly interactive polymorphisms reached statistical significance in predicting the incidence of NPC (data not shown).

## Discussion

Because NPC is consistently associated with EBV and its incidence varies depending on the geographic location, genetic variants in innate immunity-related recognition pathways may contribute to disease pathogenesis. Here, we evaluated for the first time the influence of human genetic variation in some key host antiviral sensor and antiviral receptor genes on NPC susceptibility in a North African population. Polymorphisms in the studied genes *CD209*, *DDX58*, *MBL2*, *TLR2*, *TLR3*, and *TLR9* have been reported to influence a number of infectious diseases, including HIV-1 (Koizumi *et al.* 2007; Pine *et al.* 2009), cytomegalovirus (Mezger *et al.* 2008), tuberculosis (Vannberg *et al.* 2008; Velez *et al.* 2010), hepatitis C virus (Koutsounaki *et al.* 2008; Ryan *et al.* 2010), and dengue virus (Sakuntabhai *et al.* 2005; Acioli-Santos *et al.* 2008; Wang *et al.* 2011) infection among others, revealing their potential role in host defense against pathogens.

Our genetic data from the SNP analyses pointed to the possible involvement of genetic variants within the *TLR3* gene (rs3775291/Leu412Phe) but also in the *CD209* gene (rs7248637/3′ UTR) and in the *DDX58* gene (rs56309110/promoter). The other SNPs did not show any significant association.

The most significant association with NPC risk was identified by TLR3_rs3775291. The observed 1.49-fold increase in NPC risk is modest, however; this is the magnitude of risk that one would anticipate for a heterogeneous genetic disease. Previously, several studies have suggested that the TLR3_rs3775291 variant allele plays an important role in viral infections (Yang *et al.* 2012; Dhiman *et al.* 2008; Gorbea *et al.* 2010). However, the only study so far in NPC did not find any association between this SNP and the risk of NPC in a Cantonese population (He *et al.* 2007). TLR3 recognizes double-stranded RNA and is a major effector of the immune response to viral pathogens. In addition to an antiviral interferon response (Oshiumi *et al.* 2003), it also triggers pro-apoptotic pathway by activating nuclear factor-κB (Salaun *et al.* 2006). In humans, it is expressed not only in immune cells but also in many different types of malignant cells, such as breast cancer (Gonzalez-Reyes *et al.* 2010) and melanoma cells (Salaun *et al.* 2007). EBV-encoded small, noncoding RNA (EBER) molecules exist abundantly in EBV-infected cells. They can give rise to double-stranded RNA-like structures, and induce TLR3-mediated signaling (Iwakiri *et al.* 2009). TLR3_rs3775291 is causing an amino acid change Leu412Phe, which is located next to a glycosylated asparagine at position 413, which is located within the ligand-binding surface required for receptor activation (Bell *et al.* 2005; Sun *et al.* 2006). In fact, the TLR3_rs3775291 variant allele has been reported to impair poly(I:C)-mediated NF-κB and interferon activity in transfected HEK 293T and NK cells and to affect surface TLR3 expression (Ranjith-Kumar *et al.* 2007; Gorbea *et al.* 2010; Yang *et al.* 2012). Thus, the TLR3_rs3775291 variant allele may affect the recognition of EBER, which can lead to an inhibition of apoptosis or to an EBV immunoescape and therefore enhanced risk of NPC. TLR3_rs3775291 may also have clinical importance, since TLR3 agonists have been implemented as adjuvant therapy in clinical trials for different types of cancer and therapeutic response may depend on TLR3 status of the tumor tissue (Laplanche *et al.* 2000; Salaun *et al.* 2007).

DC-SIGN is a transmembrane lectin receptor on dendritic cells (DC), which can recognize many pathogens and modulate multiple immune functions (Zhou *et al.* 2006). EBV has been observed to infect DC-SIGN−positive cells such as immature DCs, monocytes and some macrophages (Li *et al.* 2002; Severa *et al.* 2012). The only study investigating the association of polymorphisms of *CD209* with NPC risk is a study by Xu *et al.* (2010). They investigated SNPs in the promoter and found that the GG genotype of rs2287886, the AA genotype of rs735240, and the G allele of rs735239 were associated with an increased NPC risk. We did not observe any association with the promoter SNPs; however, the 3′-UTR SNP rs7248637 was associated with a reduced risk. Nothing is known about the biological significance of this variant. We can postulate that by affecting the miRNA-mediated regulatory function (FuncPred), this SNP may interfere with miRNA target recognition and lead to the reduced risk observed in the current study.

In addition to TLRs and DC-SIGN, RIG-I also can induce a DC response to viral infection (Kawai and Akira 2006). In EBV-infected Burkitt’s lymphoma cells, the EBER molecule is recognized by RIG-I, leading to activation of type I interferon signaling (Samanta *et al.* 2006). It has been shown that the innate immune response of human DCs to infection by different viruses is strongly dependent on the level of DDX58 expression, which is modified by a common polymorphism rs10813831 in *DDX58* (Hu *et al.* 2010). Still, there are hardly any case-control studies (Haralambieva *et al.* 2011). In our study, there was no association between the functional SNP rs10813831 and the risk of NPC. Instead, the G allele of DDX58_rs56309110 polymorphism was associated with a decreased risk of developing NPC. The biological function of this promoter SNP is unknown. According to FuncPred, this SNP is changing the binding site of several transcription factors, however, without any predicted functional consequences.

The present study has both strengths and limitations. The detailed clinical evaluation and the genetic homogeneity of the study population, representing two North African populations with a sufficient size, is the main strength of the current study. The fact that we selected potentially functional SNPs to our study may have increased our ability to identify SNPs related to NPC. On the other hand, because no data were available on SNP frequencies in any North African population, we used data on the CEU and the YRI populations in our selection process. As also shown by our genotyping, the genetic constitution of the Moroccan and the Tunisian population is very similar, and it has been influenced by both European and Sub-Saharan gene flow (Bosch *et al.* 2001; Hajjej *et al.* 2006). However, we may have missed some SNPs private to the North African populations. There may also be some rare SNPs with minor frequency allele <10% or SNPs with still-unknown regulatory properties that were not covered by our study. Functional analyses may contribute to the understanding of the role of the studied genes in NPC and may overcome the limitation of function prediction tools that are mostly based on sequence similarities.

In summary, our results suggest a potential role for the host genetic background in NPC susceptibility. The available case and control samples from Morocco and Tunisia provided a unique possibility to analyze the genetic background of the EBV-related cancer NPC in a high-incidence population. Polymorphisms in *CD209*, *DDX58*, and *TLR3* were associated with the risk of NPC with *TLR3*_rs3775291 showing the strongest association. Furthermore, the risk increased with increasing number of the risk alleles. Admittedly, further studies are needed to confirm our findings and to evaluate the function of the disease-associated SNPs.

## Supplementary Material

Supporting Information
