# Supplementary material for: Genetic Polymorphisms in Host Innate Immune Sensor Genes and the Risk of Nasopharyngeal Carcinoma in North Africa
Source: G3 (Bethesda). 2013 Jun 1;3(6):971–7. doi: 10.1534/g3.112.005371 (PMC3689808; doi:10.1534/g3.112.005371)
Supplement: Supporting Information [file supp_3_6_971__index.html]

Genetic Polymorphisms in Host Innate Immune Sensor Genes and the Risk of Nasopharyngeal Carcinoma in North Africa — Supporting Information 

# Genetic Polymorphisms in Host Innate Immune Sensor Genes and the Risk of Nasopharyngeal Carcinoma in North Africa

## Supporting Information for Moumad *et al.*, 2013

**Files in this Data Supplement:**

- Figure S1 - Haploview figures (PDF, 320 KB)
