# Supplementary material for: Genetic Polymorphisms in Host Innate Immune Sensor Genes and the Risk of Nasopharyngeal Carcinoma in North Africa
Source: G3 (Bethesda). 2013 Jun 1;3(6):971–7. doi: 10.1534/g3.112.005371 (PMC3689808; doi:10.1534/g3.112.005371)
Supplement: Supporting Information [file supp_g3.112.005371_FigureS1.pdf]

G3 · Genes | Genomes | Genetics

Genetic Polymorphisms in Host Innate Immune Sensor Genes and the Risk of Nasopharyngeal Carcinoma in North Africa

Khalid, Moumad*[,§,†]; Jesus, Lascorz*; Melanie, Bevier*; Meriem, Khyatti[§]; Moulay Mustapha, Ennaji[†]; Abdellatif, Benider[‡]; Stefanie, Huhn*; Shun, Lu*; Lotfi, Chouchane**; Marilys, Corbex[§§]; Kari, Hemminki*[,††] Asta, Försti*[,††]

* Department of Molecular Genetic Epidemiology, German Cancer Research Center (DKFZ), Heidelberg, Germany
[§] Oncovirology Laboratory, Institut Pasteur du Maroc, Casablanca, Morocco
[†] Laboratory of Virology, Hygiene & Microbiology, Faculty of Sciences & Technics, University Hassan II Mohammedia-Casablanca, Morocco
[‡] Service de Radiothérapie, Centre d'oncologie Ibn Rochd, Casablanca, Marocco
** Genetic Medicine and Immunology Laboratory, Weill Cornell Medical College in Qatar, Qatar Foundation, Education City, Doha, Qatar
[§§] Department of Public health, Institute of Tropical Medicine, Antwerp, Belgium
[††] Center of Primary Health Care Research, Clinical Research Center, Lund University, Malmö, Sweden

*Corresponding author:
Asta Försti, German Cancer Research Center (DKFZ), Department of Molecular Genetic Epidemiology (C050), Im Neuenheimer Feld 580, 69120 Heidelberg, Germany, Tel. +49-6221-421803, Fax +49-6221-421810, e-mail a.foersti@dkfz.de

DOI: 10.1534/g3.112.005371

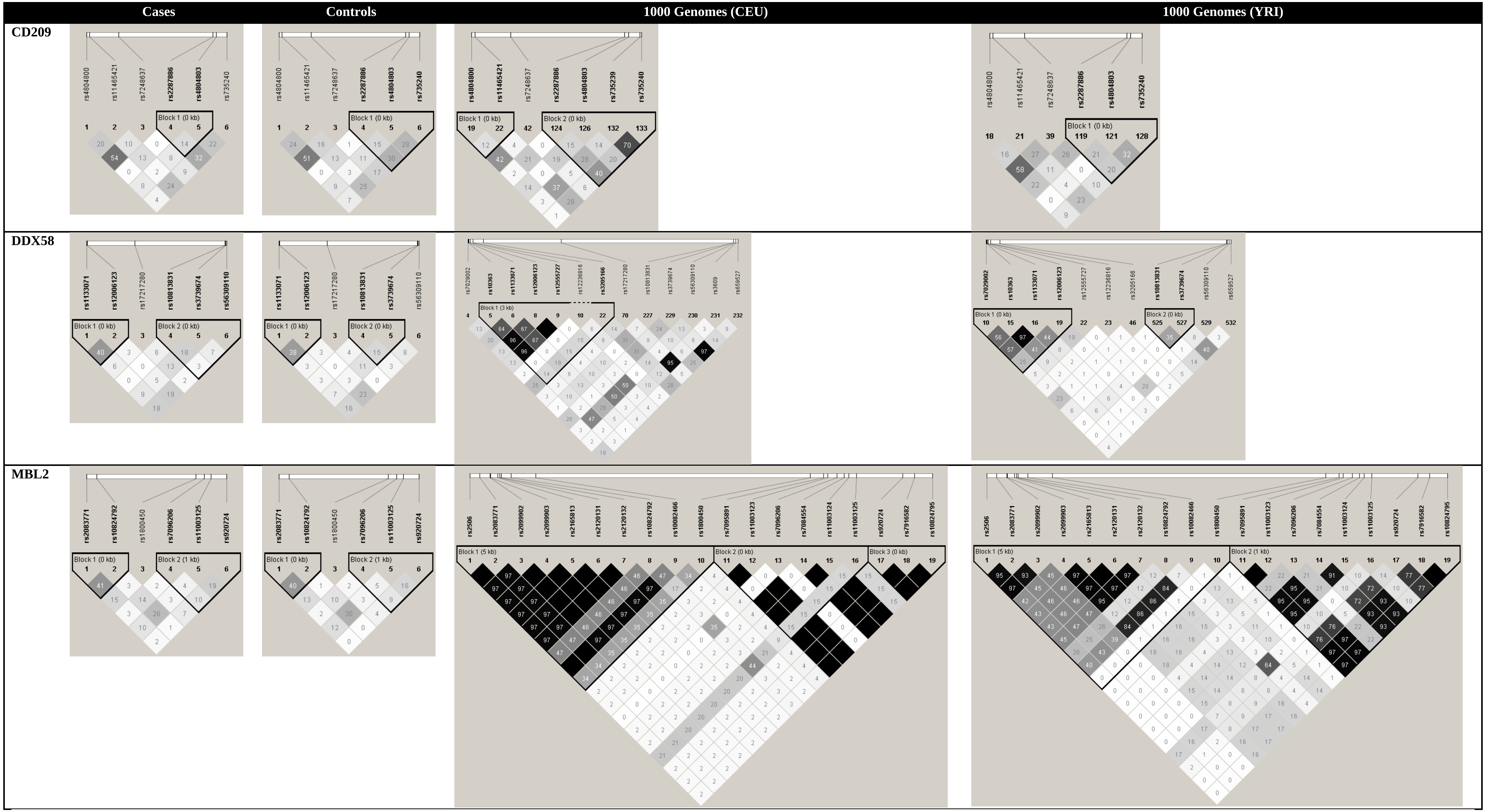

Figure S1 Haploview figures. Linkage disequilibrium (LD) plots characterizing haplotype blocks in the CD209, MBL2 and DDX58 genes. For each gene, from left to right, the LD plots for the NPC cases, the NPC controls, the CEU population and the YRI population (http://www.1000genomes.org/ensembl-browser) are shown. The genotyped SNPs and other potentially functional SNPs captured by the genotyped SNPs are shown. r^2 values are indicated in percentages within squares in the LD plot. Strong LD is indicated by dark gray, while light gray and white indicate uninformative and low confidence values, respectively. The haplotype blocks were created using HaploView program, version 4.2.
